# Re-development of mental health first aid guidelines for supporting Aboriginal and Torres Strait Islanders who are engaging in non-suicidal self-injury

**DOI:** 10.1186/s12888-017-1465-1

**Published:** 2017-08-22

**Authors:** Gregory Armstrong, Natalie Ironfield, Claire M. Kelly, Katrina Dart, Kerry Arabena, Kathy Bond, Anthony F. Jorm

**Affiliations:** 1 Centre for Mental Health, Melbourne School of Population and Global Health, The University of Melbourne, 207 Bouverie St, Carlton, Victoria 3010 Australia; 2 Mental Health First Aid Australia, Level 6, 369 Royal Parade, Parkville, Victoria 3053 Australia; 3 Indigenous Health Equity Unit, Melbourne School of Population Health, University of Melbourne, 207 Bouverie St, Carlton, Victoria 3010 Australia

**Keywords:** Non-suicidal self-injury, Indigenous, Mental health first aid, Early intervention, Helping behaviour, Assistance

## Abstract

**Background:**

Non-suicidal self-injury (NSSI) disproportionally affects Indigenous Australians. Friends, family and frontline workers (for example, teachers, youth workers) are often best positioned to provide initial assistance if someone is engaging in NSSI. Culturally appropriate expert consensus guidelines on how to provide mental health first aid to Australian Aboriginal and Torres Strait Islanders who are engaging in NSSI were developed in 2009. This study describes the re-development of these guidelines to ensure they contain the most current recommended helping actions.

**Methods:**

The Delphi consensus method was used to elicit consensus on potential helping statements to be included in the guidelines. These statements describe helping actions that Indigenous community members and non-Indigenous frontline workers can take, and information they should have, to help someone who is engaging in NSSI. The statements were sourced from systematic searches of peer-reviewed literature, grey literature, books, websites and online materials, and existing NSSI courses. A panel was formed, comprising 26 Aboriginal and Torres Strait Islanders with expertise in NSSI. The panellists were presented with the helping statements via online questionnaires and were encouraged to suggest re-wording of statements and any additional helping statements that were not included in the original questionnaire. Statements were only accepted for inclusion in the guidelines if they were endorsed by ≥90% of panellists as essential or important.

**Results:**

From a total of 185 statements shown to the expert panel, 115 were endorsed as helping statements to be included in the re-developed guidelines.

**Conclusions:**

A panel of Aboriginal and Torres Strait Islander people with expertise in NSSI were able to reach consensus on appropriate strategies for providing mental health first aid to an Aboriginal and Torres Strait Islander engaging in NSSI. The re-development of the guidelines has resulted in more comprehensive guidance than the earlier version. The re-developed guidelines will form the basis of an Aboriginal mental health first aid short course on NSSI for Indigenous community members and non-Indigenous frontline workers that will be evaluated in an upcoming trial.

**Electronic supplementary material:**

The online version of this article (doi:10.1186/s12888-017-1465-1) contains supplementary material, which is available to authorized users.

## Background

Non-suicidal self-injury (NSSI) refers to injuries that are deliberately self-inflicted and not intended to result in death [1]. The bulk of what is known today about NSSI is from studies that focus on Caucasian populations. NSSI most commonly begins in early adolescence, although it also occurs in adults, and the most frequently reported self-injury methods are skin cutting, hitting and burning [2]. Behavioural scientists are attempting to understand the function of NSSI to inform intervention strategies, and at present there is a paucity of evidence-based treatments [3–5]. Survey findings indicate that internal motivations, such as managing difficult emotions and punishing oneself, are among those reasons most frequently reported for engaging in NSSI [6]. Contrary to popular belief, self-injury is rarely used as a means of seeking attention, and self-inflicted cuts and wounds are likely to be covered or hidden.

The high rates of Indigenous suicide deaths plaguing several postcolonial countries, including Australia, Canada, the United States and New Zealand have deservedly brought increased attention to the area of deliberate self-harm [7–9]. However, the focus of research on deliberate self-harm among Indigenous peoples has been almost exclusively on behaviours with suicidal intent, with minimal attention given to behaviours with no suicidal intent. This is somewhat surprising, considering that rates of suicidal ideation and suicide attempts are far higher among those who have previously engaged or currently engage in NSSI [2, 10]. For example, Nock et al. [2] reported that 70% of adolescents engaging in NSSI reported a lifetime suicide attempt and 55% reported multiple attempts. The same association between NSSI and suicide attempts was observed in a study of American Indians of the White Mountain Apache Tribe, but there is otherwise a lack of research on NSSI among Indigenous populations [11].

In Australia, the national NSSI prevalence figures come from The Australian National Epidemiological Study of Self-Injury (ANESSI) survey, which found that the 12-month and lifetime prevalence of NSSI among a small sub-sample of Aboriginal and Torres Strait Islander people (*n* = 156, 1.7% of total sample) was 4.8% and 17.2% respectively, compared to 2.6% and 8.1% for non-Indigenous Australians [1]. The difference in rates of deliberate self-harm between Aboriginal and Torres Islander people and non-Indigenous Australians has been observed to be particularly pronounced in some regional areas; for example, rates of hospitalisation for deliberate self-harm (including self-harm with and without suicidal intent) in the Kimberley have been observed to be up to ten times higher for Aboriginal and Torres Strait Islander people when compared to international figures of hospitalisation for deliberate self-harm [12].

Friends, family and frontline workers (for example, teachers and sports coaches) are often well positioned to provide initial assistance to individuals who engage in NSSI. Although available data indicates that NSSI is a relatively common behaviour among Aboriginal and Torres Strait Islander people, there is limited information available regarding help-seeking behaviours or about how best to support an Aboriginal or Torres Strait Islander person who is engaging in NSSI. Studies conducted with predominantly Caucasian populations suggest that many individuals who engage in NSSI do not seek help [13], with Australian figures highlighting that less than 40% of individuals who engage in NSSI report help seeking behaviours [1]. Available literature indicates that when help is sought, it is primarily sought from friends and family [14–16]. As friends and family are often approached first when individuals seek support, they have the potential to play a critical role in connecting individuals with appropriate professional help and community support.

‘Mental health first aid’ is defined as the help provided to a person developing a mental health problem, experiencing the worsening of an existing mental health problem or in a mental health crisis, until appropriate professional treatment is received or until the crisis resolves [17]. In 2001, a mental health first aid training program was established in Australia in response to the need for public education about mental illness and its treatment [18]. Later, an Aboriginal and Torres Strait Islander Mental Health First Aid (AMHFA) program was established [19]. The first edition of the AMHFA course was based around a cultural adaptation of the Standard Mental Health First Aid course guided by an Indigenous working group. Subsequently, a second edition was produced based on a series of guideline documents that were developed using Delphi expert consensus studies with Aboriginal or Torres Strait Islander mental health professionals as expert panellists [20]. Based on this series of guideline documents, the AMHFA program sought to provide recommendations as to how to provide initial assistance to an Aboriginal or Torres Strait Islander person with a mental health problem or in a mental health crisis, including depression, psychosis, substance use, or experiencing a traumatic event, a panic attack, suicidal thoughts or engaging in NSSI. AMHFA guidelines were also developed around ‘*Cultural Considerations and Communication Techniques’* and ‘*Communicating with an Aboriginal or Torres Strait Islander Adolescent*’ [20, 21].

The AMHFA program is run through Mental Health First Aid Australia (MHFAA) who use a train-the-instructor style model, whereby they train their pool of accredited AMHFA Instructors, who are Aboriginal or Torres Strait Islander people, in how to deliver the course material to Indigenous community members and frontline workers (for example, teachers, youth workers) in their respective communities, where they are already embedded and have local support. An initial evaluation of the AMHFA program based on roll-out and qualitative data obtained from focus group discussions found the program to be both culturally appropriate and acceptable to Aboriginal and Torres Strait Islander people [19].

As part of the process outlined above, AMHFA guidelines for assisting an Aboriginal or Torres Strait Islander person engaging in NSSI were first developed in 2009. The aim of this current study was to use the Delphi methodology to re-develop these guidelines in order to ensure that they reflect current evidence and best practice in NSSI prevention, and contain the most current recommended helping actions that can be shared with Aboriginal and Torres Strait Islander community members and non-Indigenous frontline workers. Further, this study aimed to expand upon previous guidelines and provide more comprehensive guidance as to how members of the public can provide mental health first aid to an Aboriginal or Torres Strait Islander person engaging in NSSI. The re-development of these guidelines will inform the development of an AMHFA training course on NSSI.

## Methods

The Delphi consensus method has been used extensively in health and social research as a method for decision-making processes, including mental health research [22]. The Delphi method provides a platform for obtaining expert consensus on what constitutes best practice in scenarios that cannot be feasibly or ethically subject to a randomised controlled trial. The process involves a series of questionnaires being sent to a group of experts, who do not have to attend group meetings and can respond anonymously. Traditionally, the Delphi method has involved a number of iterations before consensus is achieved. Feedback is given at each stage in order to help experts assess their opinions against those of the group.

We used the Delphi consensus method to elicit consensus on potential helping statements to be included in the guidelines. The development of the guidelines using the Delphi method involved four steps: 1) formation of the expert panel, 2) questionnaire development, 3) data collection and analysis, and 4) guideline development.

### Panel formation

A panel was recruited, comprising 26 Aboriginal and Torres Strait Islander people who had expertise in deliberate self-harm (both with and without suicidal intent) through their professional experience. A recruitment advertisement was sent out via the Aboriginal Mental Health First Aid Instructor email list, the Onemda VicHealth Koori Health Unit (University of Melbourne) email list, and the Lowitja Institute email list. The advertisement encouraged people to distribute the flyer across their broader networks. Potential candidates were asked to contact the research coordinator with information on their professional experience and were sent a Plain Language Statement prior to participation. The research was approved by the Human Research Ethics Sub-Committee at the University of Melbourne (HREC No.1443056.1). Expert panel members were reimbursed AUD$250 for completing all three survey rounds.

### Questionnaire development

The questionnaire contained statements describing helping actions that Indigenous community members and non-Indigenous frontline workers can take, and information they should have, to help an Aboriginal or Torres Strait Islander person who is engaging in NSSI. Statements were considered acceptable for inclusion in the questionnaire if the working group (comprising the authors) agreed that they described how someone can help a person who is engaging in NSSI with clear and non-ambiguous actions.

The statements were sourced from two previous Delphi questionnaires; the first questionnaire was designed to develop the original Aboriginal mental health first aid guidelines for NSSI in 2009 and the second questionnaire was designed to re-develop the mainstream mental health first aid guidelines for NSSI in 2014 [20, 23]. These questionnaires were formed through systematic searches of peer-reviewed literature, grey literature, books, websites and online materials, and existing NSSI courses, and these literature searches are described in detail elsewhere [20, 23]. The statements in the questionnaire were divided into eight sections based on common themes. The statements derived from the literature were kept as intact as possible to remain faithful to the original wording of the information. Statements were only modified to ensure consistency of format, or where there was concern about the comprehensibility or cultural appropriateness of the information.

### Data collection and analysis

Once panel members had been recruited, they were sent an electronic link to an online questionnaire hosted by Survey Monkey. Participants responded by rating how important the first aid action statements were to the development of a set of guidelines on providing mental health first aid to an Aboriginal or Torres Strait Islander person who is experiencing NSSI. Each statement was rated using a five-point scale with the following options: *Essential, Important, Don’t know/It depends, Unimportant, Should not be included*.

Pre-determined criteria were used to assess the outcome for each statement. Statements were immediately included in the guidelines if they were endorsed by ≥90% of panellists as either *Essential* or *Important*. Statements were re-rated in the Round 2 questionnaire if they were rated as *Essential* or *Important* by 80–89.9% of the panel. Statements were immediately excluded from the guidelines if they were rated as *Essential* or *Important* by less than 80.0% of both panel members.

In Round 1, panel members were also invited to make comments on any ambiguity or wording of the statements presented, and to suggest new statements that had not yet been considered, through a feedback textbox at the end of each section of the questionnaire. The comments made were reviewed by the working group. Suggestions that contained novel ideas were used to create new helping statements to be included in the subsequent Round 2 questionnaire. Statements that received comments suggesting ambiguity in the interpretation of its meaning were re-phrased to make them clearer and were also included in the Round 2 questionnaire. Round 2 also included any statements from Round 1 that needed to be re-rated.

A Round 3 questionnaire would often be used in Delphi studies where necessary, and would be comprised of any new statements that were developed from Round 1 feedback and had been presented for the first time in Round 2, but required re-rating in a further round. In our study, there were no items that were introduced in Round 2 that required re-rating in a Round 3 questionnaire; thus, the study was complete after two rounds of questionnaires.

Following each round of the two rounds of questionnaires, each panellist was sent a report containing a summary of the results from the previous round, with the report personalised to include the individual panellist’s rating for each statement, as well as a table summary of the overall panel’s rating for the statement. This allowed the panellists to compare their ratings with the level of endorsement given by the group as a whole and to inform their future ratings for those statements that needed to be re-rated.

### Guideline development

All statements endorsed as either *Essential* or *Important* by ≥90% of the panel members were written into a guideline document. One author (NI) drafted the guidelines by writing the list of endorsed statements into sections of prose based on common themes. Where possible, statements were combined and repetition deleted to reduce length. The draft was then presented to the working group, who edited the document to create a set of guidelines that were written in plain English and were easy to follow. A number of drafting iterations were completed before the group agreed upon the final document, a copy of which was sent to each panel member for review. While panellists could not suggest new content at this stage, they were able to provide feedback on the wording and layout of the document to improve clarity and reduce ambiguity.

## Results

### Expert panel members

We recruited 26 expert panel members (19 female, 8 male, age range 28 to 58 years), who completed the Round 1 questionnaire. Of the 26, 96.2% (*n* = 25) were retained in the study and also completed the Round 2 questionnaire. Approximately one-third (37.0%, *n* = 10) of the panel heard about the study through the Onemda VicHealth Koori Health Unit email list, 7.7% (*n* = 2) through the Aboriginal Mental Health First Aid instructor list, 7.4% (*n* = 2) through a colleague and 3.2% (*n* = 1) through the Lowitja Institute email list and 40.7% (*n* = 11) were recruited through other pathways, which is unsurprising given the advertisement encouraged people to distribute the flyer across their broader networks. The panel members came from a range of health and community services roles: 5 panel members were social workers, 6 were Aboriginal Mental Health Workers, 3 were nurses, 2 were GPs, 2 were academics, 2 were Aboriginal Health Workers, 2 were Aboriginal Mental Health Policy Advisors, 1 was an Aboriginal Community Support Worker, 1 was an Indigenous Public Health Officer and 2 were other types of health workers. Many members of the panel held multiple other community roles (for example, participation in suicide prevention evaluations), indicating a high level of community engagement.

Further socio-demographic information on the panel members is provided in Table 1. The majority of panel members identified as being Aboriginal, with one identifying as Torres Strait Islander and one identifying as both Aboriginal and Torres Strait Islander. There was a broad representation of States and Territories across Australia, with panel members from every state and territory. It is important to note that while we recruited a panel of people with professional expertise, almost all panel members reported also having had personal experience (outside of their professional role) with NSSI in either themselves, their families, their friends, or in their broader community network. Just one panel member had not had any personal experience with NSSI. This indicates that almost all panel members were able to draw on both professional and personal experiences when rating the statements in the questionnaires, adding an important richness to their expertise.

**Table 1:** Characteristics of panel members (*n* = 26)

	% (n)
Age group (range: 28–58, mean: 45.5 years)	
25–40 years	19.2% (5)
41–50 years	46.2% (12)
51–60 years	34.6% (9)
Gender	
Female	69.2% (18)
Male	30.8 (8)
Indigenous identification	
Aboriginal	92.3% (24)
Torres Strait Islander	3.8% (1)
Both Aboriginal and Torres Strait Islander	3.8% (1)
State where currently working	
Victoria	23.1% (6)
Queensland	15.4% (4)
Western Australia	11.5% (3)
New South Wales	11.5% (3)
Northern Territory	7.7% (2)
South Australia	7.7% (2)
Australian Capital Territory	7.7% (2)
Tasmania	3.8% (1)
Australia wide	11.5% (3)
Personal (i.e. not professional) experience with non-suicidal self-injury	
In myself	16.0% (4)
In my family	64.0% (16)
In my friends	52.0% (13)
In my broader community network	64.0% (16)
No personal experience	4.0% (1)
I’d rather not say	0.0% (0)

### Ratings of the statements

**Fig. 1:**
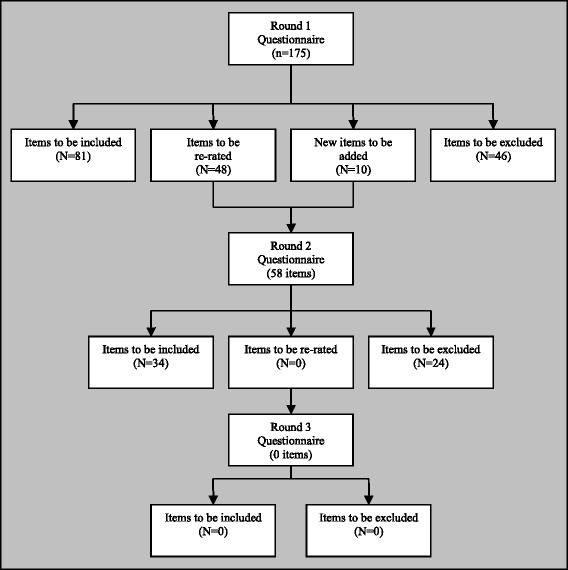
Overview of the three rounds of the Delphi method

An overview of the three rounds of the Delphi study is provided in Fig. 1 and a breakdown of the number of endorsed and rejected items for each section of the Delphi questionnaire is provided in Table 2. We started with a total of 175 statements in the Round 1 questionnaire, and included an additional 10 new statements based on feedback from the panel, resulting in a total of 185 different statements being rated by the panel across the two rounds of questionnaires. Of these 185 statements, 115 were endorsed as being either *Important* or *Essential* for the guidelines by ≥90% of panellists; 81 statements were endorsed in Round 1 and 34 in Round 2. A total of 70 statements were not endorsed for the guidelines; 46 statements were rejected in Round 1 and 24 in Round 2. Additional file 1 contains the ratings for all the statements (both endorsed and rejected) that were presented to the panel.

**Table 2:** Sections in the Delphi questionnaire, and the number of items endorsed and rejected

Section	Topic	Number of items endorsed	Number of items rejected	Total
1	What the first aider should know about self-injury	17	11	28
2	If the first aider finds someone injuring themselves	11	8	19
3	If the first aider suspect self-injury	6	8	14
4	Discussing self-injury	44	22	66
5	Alternatives to self-injury	14	8	22
6	Harm minimisation practices	1	4	5
7	Seeking help	13	2	15
8	Adolescent specific	9	7	16
	TOTAL	115	70	185

## Discussion

The aim of this study was to re-develop the Aboriginal mental health first aid guidelines for members of the public in providing assistance to an Aboriginal or Torres Strait Islander person engaging in NSSI. This was achieved by drawing on the knowledge of a panel of Aboriginal and Torres Strait Islander people who have expertise in NSSI. Despite being from diverse backgrounds and geographical locations across Australia, the expert panel was able to reach a high level of consensus on a range of mental health first aid techniques, and 115 statements that were endorsed by ≥90% of panellists were included in the re-developed guidelines.

### Comparison with the original Aboriginal mental health first aid guidelines for NSSI

The re-development of the guidelines has resulted in more comprehensive guidance than the earlier version. For the development of the original version, panellists had rated 114 helping statements and had endorsed 31 statements. For our re-developed version, panellists rated 185 statements and endorsed 115 statements. The increase in the number of statements rated by panellists and included in the guidelines is a reflection of the growth of NSSI expertise and advice available in the published literature, grey literature, on websites, and other sources. This highlights the importance of conducting revisions of guideline documents, as the advice provided by the literature and expert opinion can change across the span of a few years.

The re-developed guidelines reaffirmed some important cultural elements of the original guidelines that are important for people to consider when assisting an Aboriginal or Torres Strait Islander person who is engaging in NSSI. The importance of community norms and cultural context was again prominent in the re-developed guidelines, with the endorsement of statements such as the first aider should: “*learn about the behaviours that are considered warning signs for self-injury in the person’s community*”; “*take into consideration the spiritual and/or cultural context of the person’s self-injury*”; “*be aware that pathological self-injury, such as cutting and burning, is fundamentally different to ritualistic, culturally accepted Aboriginal ceremonial or grieving practice*”; and, should “*distinguish between cultural practice and deliberate self-injury by engaging the help of local Aboriginal health workers or respected Elders*”. Several statements also noted the need for first aiders to consider that there may be a broad range of potential community supports that may be preferred as sources of support by Aboriginal and Torres Strait Islander people, for example, family and friends, Aboriginal health workers and community liaison officers.

### Comparison with the mainstream (i.e. non-Indigenous specific) mental health first aid guidelines for NSSI

There were 141 statements that appeared in both the first round of the current Delphi study and a 2014 Delphi study designed to develop the mainstream (i.e. not specific to Aboriginal and Torres Strait Islander people) mental health first aid guidelines for NSSI. The 2014 Delphi study had two panels, a consumer panel and a professional panel, whereas the current study only had a professional panel (almost all of whom also had personal exposure to NSSI). The ratings given by our Aboriginal and Torres Strait Islander panel were, on average, statistically significantly higher than those ratings given by the mainstream professional panel in 2014, but there was no significant difference in average ratings with the mainstream consumer panel. On average, 85.4% of our Aboriginal and Torres Strait Islander panel rated each of the comparable statements as either important or essential for the AMHFA suicide guidelines, compared to an average of 75.9% (t(278) = 4.8, *p* < 0.001) of professionals and 83.5% (t(278) = 1.1, *p* = 0.281) of consumers in the 2014 mainstream Delphi study.

Among those statements rated in both studies, the endorsement ratings given by our Aboriginal and Torres Strait Islander panel were strongly correlated across items with both the consumer panel r(138) = 0.73, *p* < 0.05 and the professional panel r(138) = 0.71, *p* < 0.05 from the 2014 mainstream Delphi study. In terms of comparing whether statements were endorsed or not between the two Delphi studies for each of the 140 statements, there was a moderate level of inter-rater agreement with a kappa co-efficient of 0.58. In practical terms, the two Delphi studies came to the same conclusion about endorsing or not-endorsing an item on 80.7% of occasions; 86.4% of statements endorsed in the 2014 mainstream Delphi were also endorsed in the current study and 71.2% of statements rejected in the 2014 mainstream Delphi study were also rejected in the current study. The strongly correlated ratings across the two Delphi studies suggests a high degree of overlap in terms of knowledge of NSSI between the panel of Indigenous professional experts and the non-Indigenous consumer and professional panels, in relation to the sub-set of statements that were presented to both the current and former panels. The high proportion of these statements that were endorsed by both panels indicates a moderate degree of transferability of action statements between the mainstream guidelines and the Aboriginal mental health first aid NSSI guidelines.

### Important considerations when using the guidelines

The specific purpose of the guidelines developed through this study is to inform the actions undertaken by mental health first aiders, and the guidelines will be used by MHFAA to revise the curriculum of the AMHFA course to a third edition and to develop a new Indigenous specific NSSI training course to be rolled out by their AMHFA Instructors.

Nonetheless, the guidelines may be useful to others working with Aboriginal and Torres Strait Islander people who engage in NSSI. The guidelines make many important and useful recommendations to be considered by those offering support to an Aboriginal or Torres Strait Islander person engaging in NSSI, for example: take all self-injuring behaviour seriously, regardless of the severity of the injuries or the intent; use a calm voice and reassure the person that you are not disgusted by them and that they are not a bad person; keep in mind that ‘stopping self-injury’ should not be the focus of the conversation and do not try to make the person stop self-injuring; allow for periods of silence to give the person time to process what is being talked about; work collaboratively with the person in finding solutions; acknowledge the emotional pain behind their self-injury; and, encourage the person to explore alternative strategies to relieve their distress that do not involve self-injuring. These guidelines can offer a level of confidence to users that a panel of Indigenous people with professional expertise in NSSI have endorsed these strategies.

However, it is important to consider some of the following issues before using these guidelines to inform Indigenous NSSI training programs. Firstly, the guidelines should not be used in isolation. There are other Aboriginal Mental Health First Aid Guidelines that could also be referred to, most notably the guidelines on ‘*Cultural Considerations and Communication Techniques*’, ‘*Communicating with an Aboriginal or Torres Strait Islander Adolescent*’ and ‘*Guidelines for Providing Mental Health First Aid to Aboriginal and Torres Strait Islander People Experiencing Suicidal Thoughts and Behaviour*’ [20, 21, 24]. Those implementing the recommendations as first aiders will also need other local knowledge relevant to their respective communities. Aboriginal and Torres Strait Islander communities are not homogenous and, as such, reading these generalised guidelines in isolation is unlikely to be sufficient.

Secondly, while the guidelines do offer recommendations about how an Indigenous community member or non-Indigenous frontline health worker may support an Aboriginal or Torres Strait Islander person engaging in NSSI, and what they may need to know to be able to do this, they don’t specify how programs or training courses based around these recommendations should be developed, packaged and integrated within broader community-based programs. In developing NSSI training programs based on these guidelines, it is important to consider the use of culturally appropriate frameworks for talking holistically about mental health and NSSI (for example, the concept of social and emotional wellbeing) [25, 26]. It is also important to consider a ‘whole of community’ approach that focuses on connectedness, belongingness, cultural heritage, community ownership and connections with a broader suite of community developments [25]. Additionally, creative methods of delivering some of the key messages in these guidelines could be considered: art classes, storytelling, dancing events, theatrical showcases, cultural camps and community activities may all be highly effective ways for some Indigenous communities to engage with the recommendations in these guidelines.

Thirdly, one common strategy of supporting someone engaging in NSSI is to link them with mental health services. We must acknowledge that referring Aboriginal or Torres Strait Islander people to mental health services can be problematic. There are important barriers that prevent formal mental health services from being an ideal source of care for Indigenous people which have been documented in Australia, Canada, the United States and elsewhere, for example: 1) the stigma and shame Indigenous people may experience when accessing formal mental health services; 2) experiences of discrimination and racism within the broader health system, which can in turn worsen psychological distress; 3) the provision of individualised care, rather than community- or family-based interventions, that diminishes the value that many Indigenous people place on interconnectedness; 4) concerns that formal mental health services may not be provided in a way that is compatible with the holistic nature of the social and emotional wellbeing framing of Indigenous mental health; and 5) a lack of engagement with cultural or spiritual approaches to nurturing social and emotional wellbeing (for example, community gatherings, intergenerational transmission of knowledge and stories, dancing, healing ceremonies, and nature-based activities), which are largely distanced as being outside the bounds of evidence-based mental health care [25, 27–36]. Formal mental health services are a critical resource for Aboriginal and Torres Strait Islander people engaging in NSSI, however, the challenges are many for them to become culturally safe and appropriate sources of care. Indigenous NSSI programs can acknowledge these shortcomings and work with communities to discuss and establish acceptable ways of accessing support and care from different sources, while advocating with mental health services around the need to develop holistic, flexible and culturally appropriate approaches.

### Limitations and future research

Our panel was formed entirely of Aboriginal and Torres Strait Islander people with professional experience in NSSI. Future research could consider also having a panel of Aboriginal and Torres Strait Islander people who identify as consumers of NSSI services or carers of people who have engaged in NSSI, as such people will bring a different type of equally important expertise that would have added great value to the re-development of these guidelines. However, almost all of our professional panel members also had personal exposure to NSSI in either themselves, their families, their friends, or in their broader community network, and thus had a broad range of expertise to draw upon. Future research could also endeavour to incorporate the voices and experiences of young consumers of NSSI services, among whom the prevalence of NSSI is greatest.

Even though we had sought a panel with professional expertise to help devise these guidelines, the panel members may have alternated between drawing on either their professional or personal experiences in responding to the statements and thus their responses may not be equivalent to a panel of people with only professional expertise. However, we have highlighted that the ratings given by the Aboriginal and Torres Strait Islander professional panel in this study were strongly correlated to the ratings of both the consumer and professional panels used to create the mainstream NSSI guidelines, indicating a high degree of overlap in terms of NSSI knowledge.

It must be kept in mind that the helping actions endorsed in the guidelines are based on expert opinion; these are the recommendations of experts in the absence of evidence from experimental studies about how best to provide mental health first aid to an Aboriginal or Torres Strait Islander person engaging in NSSI. Additionally, these guidelines are recommended for use by mental health first aiders only. While the actions endorsed in these guidelines may be useful in different aspects of Indigenous NSSI prevention, these are specific to the recommended support that can be provided by mental health first aiders. These guidelines take into consideration the limitations in the first aiders’ support role, and guide the first aider on how to act within these. Nonetheless, these guidelines may be useful to those working outside the scope of the mental health first aid paradigm.

The vast majority of the NSSI literature is based on studies and reports that are not specific to Aboriginal and Torres Strait Islander peoples, or other Indigenous communities in other countries. In fact, there is very little literature at all on the topic of NSSI among Indigenous people anywhere in the world. Thus, the majority of the statements presented to our panel, for endorsement or otherwise, were generated from the mainstream NSSI literature. This put a great onus on our expert panel to either suggest new culturally appropriate helping statements or to suggest re-wording of existing actions so that they were more culturally appropriate. This would be a difficult task for panel members given they were already faced with reviewing a large number of helping statements.

Finally, only two panel members identified as Torres Strait Islander, which may affect the generalisability of the findings for Torres Strait Islander peoples.

## Conclusions

Through the Delphi process, the Aboriginal mental health first aid guidelines for supporting an Aboriginal or Torres Strait Islander person engaging in NSSI have been updated to ensure they are current and include the most recent and appropriate helping actions. This re-development has added depth to the previous version of the guidelines. These guidelines will now be made freely available for download on the MHFAA website (https://mhfa.com.au) and will also be used to form the basis of an AMHFA NSSI training course aimed at educating members of the public in providing mental health first aid to an Aboriginal or Torres Strait Islander person who is engaging in NSSI.

## Additional file

Additional file 1: AMHFA NSSI endorsed and rejected items, contains the ratings for all the statements that were presented to the panel. (XLSX 21 kb)

## Abbreviations

AMHFA: Aboriginal mental health first aid
MHFAA: Mental health first aid Australia
NSSI: Non-suicidal self-injury

## References

[1] Martin G, Swannell S, Harrison J, Hazell P, Taylor A (2010). The Australian National Epidemiological Study of self-injury (ANESSI).

[2] Nock MK, Joiner TE, Gordon KH, Lloyd-Richardson E, Prinstein MJ (2006). Non-suicidal self-injury among adolescents: diagnostic correlates and relation to suicide attempts. Psychiatry Res.

[3] Gonzales AH, Bergstrom L (2013). Adolescent non-suicidal self-injury (NSSI) interventions. J Child Adolesc Psychiatr Nurs.

[4] Prinstein MJ (2008). Introduction to the special section on suicide and nonsuicidal self-injury: a review of unique challenges and important directions for self-injury science. J Consult Clin Psychol.

[5] Ougrin D, Tranah T, Stahl D, Moran P, Asarnow JR (2015). Therapeutic interventions for suicide attempts and self-harm in adolescents: systematic review and meta-analysis. J Am Acad Child Adolesc Psychiatry.

[6] Nock MK, Prinstein MJ (2004). A functional approach to the assessment of self-mutilative behavior. J Consult Clin Psychol.

[7] McLoughlin AB, Gould MS, Malone KM (2015). Global trends in teenage suicide: 2003-2014. QJM.

[8] Clifford AC, Doran CM, Tsey K (2013). A systematic review of suicide prevention interventions targeting indigenous peoples in Australia, United States, Canada and New Zealand. BMC Public Health.

[9] Armstrong G, Pirkis J, Arabena K, Currier D, Spittal MJ, Jorm AF. Suicidal behaviour in Indigenous compared to non- Indigenous males in urban and regional Australia: prevalence data suggest disparities increase across age groups. Aust N Z J Psychiatry. 2017. 10.1177/0004867417704059 28393536

[10] Muehlenkamp JJ, Gutierrez PM (2007). Risk for suicide attempts among adolescents who engage in non-suicidal self-injury. Arch Suicide Res.

[11] Cwik MF, Barlow A, Tingey L, Larzelere-Hinton F, Goklish N, Walkup JT (2011). Nonsuicidal self-injury in an American Indian reservation community: results from the White Mountain Apache surveillance system, 2007-2008. J Am Acad Child Adolesc Psychiatry.

[12] McHugh C, Campbell A, Chapman M, Balaratnasingam S (2016). Increasing Indigenous self-harm and suicide in the Kimberley: an audit of the 2005-2014 data. Med J Aust.

[13] Rowe SL, French RS, Henderson C, Ougrin D, Slade M, Moran P (2014). Help-seeking behaviour and adolescent self-harm: a systematic review. Aust N Z J Psychiatry.

[14] De Leo D, Heller T (2004). Who are the kids who self-harm? An Australian self-report school survery. MJA.

[15] Fortune S, Sinclair J, Hawton K (2008). Help-seeking before and after episodes of self-harm: a descriptive study in school pupils in England. BMC Public Health.

[16] Evans E, Hawton K, Rodham K (2005). In what ways are adolescents who engage in self-harm or experience thoughts of self-harm different in terms of help-seeking, communication and coping strategies?. J Adolesc.

[17] Kitchener BA, Jorm AF, Kelly CM (2015). Mental health first aid international manual.

[18] Kitchener BA, Jorm AF (2002). Mental health first aid training for the public: evaluation of effects on knowledge, attitudes and helping behavior. BMC Psychiatry.

[19] Kanowski LG, Jorm AF, Hart LM (2009). A mental health first aid training program for Australian Aboriginal and Torres Strait Islander peoples: description and initial evaluation. Int J Ment Health Syst.

[20] Hart LM, Jorm AF, Kanowski LG, Kelly CM, Langlands RL (2009). Mental health first aid for Indigenous Australians: using Delphi consensus studies to develop guidelines for culturally appropriate responses to mental health problems. BMC Psychiatry.

[21] Chalmers KJ, Bond KS, Jorm AF, Kelly CM, Kitchener BA, Williams-Tchen A (2014). Providing culturally appropriate mental health first aid to an Aboriginal or Torres Strait Islander adolescent: development of expert consensus guidelines. Int J Ment Health Syst.

[22] Jorm AF (2015). Using the Delphi expert consensus method in mental health research. Aust N Z J Psychiatry.

[23] Ross AM, Kelly CM, Jorm AF (2014). Re-development of mental health first aid guidelines for non-suicidal self-injury: a Delphi study. BMC Psychiatry.

[24] Guidelines for Providing Mental Health First Aid to Aboriginal and Torres Strait Islander People Experiencing Suicidal Thoughts and Behaviour. https://mhfa.com.au/sites/default/files/AMHFA_Suicide_guidelines_inhouse%20print.pdf.

[25] Ridani R, Shand FL, Christensen H, McKay K, Tighe J, Burns J, Hunter E (2015). Suicide prevention in Australian Aboriginal communities: a review of past and present programs. Suicide Life Threat Behav.

[26] Aboriginal and Torres Strait Islander Suicide Prevention Evaluation Project (2016). Solutions that work: what the evidence and our people tell us.

[27] Farrelly T (2008). The aboriginal suicide and self-harm help-seeking quandary. Aborig Islander Health Worker J.

[28] Kelaher MA, Ferdinand AS, Paradies Y (2014). Experiencing racism in health care: the mental health impacts for Victorian Aboriginal communities. Med J Aust.

[29] Wexler LM, Gone JP (2012). Culturally responsive suicide prevention in indigenous communities: unexamined assumptions and new possibilities. Am J Public Health.

[30] Wexler L, White J, Bridie T (2016). Why an alternative to suicide prevention gatekeeper training is needed for rural Indigenous communities: presenting an empowering community storytelling approach. Critical Public Health.

[31] Battiste K (2002). Indigenous knowledge and pedagogy in first nations education: a literature review with recommendations.

[32] Isaacs AN, Pyett P, Oakley-Browne MA, Gruis H, Waples-Crowe P (2010). Barriers and facilitators to the utilization of adult mental health services by Australia's Indigenous people: seeking a way forward. Int J Ment Health Nurs.

[33] Fielke K, Cord-Udy N, Buckskin J, Lattanzio A (2009). The development of an 'Indigenous team' in a mainstream mental health service in South Australia. Australas Psychiatry.

[34] Hepworth J, Askew D, Foley W, Duthie D, Shuter P, Combo M, Clements LA (2015). How an urban Aboriginal and Torres Strait Islander primary health care service improved access to mental health care. Int J Equity Health.

[35] Kirmayer LJ (2012). Cultural competence and evidence-based practice in mental health: epistemic communities and the politics of pluralism. Soc Sci Med.

[36] McKenna B, Fernbacher S, Furness T, Hannon M (2015). "cultural brokerage" and beyond: piloting the role of an urban aboriginal mental health liaison officer. BMC Public Health.

